# Supplementary material for: The genetic architecture of human cerebellar morphology supports a key role for the cerebellum in human evolution and psychopathology
Source: Commun Biol. 2026 Feb 17;9:445. doi: 10.1038/s42003-026-09664-1 (PMC13021948; doi:10.1038/s42003-026-09664-1)
Supplement: Supplementary file 3 — Description of Additional Supplementary files [file 42003_2026_9664_MOESM3_ESM.pdf]

Description of Additional Supplementary files

Supplementary Data 1. Overlap between NNMF-derived cerebellar components and standard cerebellar anatomical regions (lobules)

Supplementary Data 2. Overlap between NNMF-derived cerebellar components and functionally defined cerebellar regions

Supplementary Data 3. LD-score regression based genetic correlations between comparison brain phenotypes in the current study and previously published GWAS results

Supplementary Data 4. LD-score regression based genetic correlations between cerebellar morphological features analyzed in the discovery and replication samples

Supplementary Data 5. GCTA-based heritability estimates for cerebellar morphological features and cerebral comparison phenotypes

Supplementary Data 6. GCTA-based bivariate genetic correlation estimates for cerebellar morphological features

Supplementary Data 7. Bivariate correlations between regional gene expression profiles for the cerebellar morphological features extracted from the Allen Human Brain Atlas

Supplementary Data 8. Functional annotation of single nucleotide polymorphisms (SNPs) in linkage disequilibrium ($r2 \geq 0.6$) with one of the independent significant SNPs associated with cerebellar morphology (multivariate GWAS; CER)

Supplementary Data 9. Functional consequences of SNPs on genes

Supplementary Data 10. Genetic loci associated with cerebellar morphology (multivariate GWAS; CER)

Supplementary Data 11. Evolutionary age distribution of candidate SNPs for CER

Supplementary Data 12. Evolutionary age distribution of candidate SNPs for cerebral comparison phenotypes (hippcampus, cerebrocortical area, cerebrocortical thickness)

Supplementary Data 13. Evolutionary age distribution of independent SNPs for cerebellar and cerebrocortical morphology

Supplementary Data 14. Genome-wide gene-based association analyses (MAGMA) for CER

Supplementary Data 15. Regional Brain Tissue Gene Expression Analysis for CER (MAGMA continuous gene-property analysis)

Supplementary Data 16. Developmental Brain Tissue Gene Expression Analysis for CER (MAGMA continuous gene-property analysis)

Supplementary Data 17. Associations between human accelerated (HAR) genes and CER and comparison cerebral phenotypes (MAGMA discrete gene-set analysis)

Supplementary Data 18. Allen Mouse Brain Atlas regional gene-expression gene-sets

significantly associated with CER (MAGMA discrete gene-set analysis)

Supplementary Data 19. Mouse gene-perturbation gene-sets significantly associated with CER (MAGMA discrete gene-set analysis)
