# Supplementary material for: The genetic architecture of human cerebellar morphology supports a key role for the cerebellum in human evolution and psychopathology
Source: Commun Biol. 2026 Feb 17;9:445. doi: 10.1038/s42003-026-09664-1 (PMC13021948; doi:10.1038/s42003-026-09664-1)
Supplement: Supplementary file 5 — nr-reporting-summary [file 42003_2026_9664_MOESM5_ESM.pdf]

nature portfolio

Corresponding author(s): Torgeir Moberget

Last updated by author(s): 05/06/2024

Reporting Summary

Nature Portfolio wishes to improve the reproducibility of the work that we publish. This form provides structure for consistency and transparency in reporting. For further information on Nature Portfolio policies, see our Editorial Policies and the Editorial Policy Checklist.

Statistics

For all statistical analyses, confirm that the following items are present in the figure legend, table legend, main text, or Methods section.

n/a	Confirmed	
☐	☒	The exact sample size (*n*) for each experimental group/condition, given as a discrete number and unit of measurement
☐	☒	A statement on whether measurements were taken from distinct samples or whether the same sample was measured repeatedly
☐	☒	The statistical test(s) used AND whether they are one- or two-sided<br>*Only common tests should be described solely by name; describe more complex techniques in the Methods section.*
☐	☒	A description of all covariates tested
☐	☒	A description of any assumptions or corrections, such as tests of normality and adjustment for multiple comparisons
☐	☒	A full description of the statistical parameters including central tendency (e.g. means) or other basic estimates (e.g. regression coefficient) AND variation (e.g. standard deviation) or associated estimates of uncertainty (e.g. confidence intervals)
☐	☒	For null hypothesis testing, the test statistic (e.g. *F*, *t*, *r*) with confidence intervals, effect sizes, degrees of freedom and *P* value noted<br>*Give P values as exact values whenever suitable.*
☒	☐	For Bayesian analysis, information on the choice of priors and Markov chain Monte Carlo settings
☒	☐	For hierarchical and complex designs, identification of the appropriate level for tests and full reporting of outcomes
☐	☒	Estimates of effect sizes (e.g. Cohen's *d*, Pearson's *r*), indicating how they were calculated

Our web collection on statistics for biologists contains articles on many of the points above.

Software and code

Policy information about availability of computer code

Data collection	No software was used for data collection in this study, as the data was acquired by the UK Biobank study team.

March 2021

Data analysis	MRI data was first processed using the recon-all pipeline in Freesurfer 5.3 (https://surfer.nmr.mgh.harvard.edu). Next, the bias-field corrected T1-images from the FreeSurfer analyses were analyzed using the cerebellum-optimized SUIT-toolbox (https://github.com/jdiedrichsen/suit). The modulated cerebellar grey matter maps from SUIT were then decomposed using orthogonal projective non-negative matrix factorization (https://github.com/asotiras/brainparts). Non-negative component weights were adjusted for effects of age, sex, estimated total intracranial volume, scanner site, 40 genetic population components, genetic batch and mean Euler number (i.e, an index of MRI image quality). Finally, all adjusted anatomical indices were inverse rank normalized. For all genetic analyses we made use of the UKB v3 imputed data, which has undergone extensive quality control procedures as described by the UKB genetics team. SNP-based heritability estimates for all morphological features – as well as the pairwise genetic correlations between cerebellar features - were estimated using genetic complex trait analysis (GCTA). Univariate GWAS analyses were conducted using Plink v1.9. Genetic correlation analyses were conducted using LD-score regression. For our main analysis, we used a recently developed multivariate analysis method (MOSTest, see more details in the Models & Analysis section below), to conduct a multivariate genome-wide association (GWA) analysis on cerebellar morphological features. MOSTest identifies genetic effects across multiple phenotypes, yielding a multivariate GWAS summary statistic across all 23 features, and provides robust (permutation based) test statistics. Locus identification and SNP annotation was performed in FUMA (https://fuma.ctglab.nl/), while we performed gene-based genome-wide analyses and gene set analyses using MAGMA (https://ctg.cncr.nl/software/magma). Regional gene expression profiles from the Allen Human Brain Atlas were extracted using the abagen toolbox (https://github.com/rmarkello/abagen). Genetic overlap with mental disorders was assessed using conjunctional FDR (https://github.com/precimed/pleiofdr/) and LD score regression (https://github.com/bulik/ldsc) analyses. The estimated evolutionary age of SNPs were downloaded rom the Human Genome Dating (HGD) website: https://human.genome.dating/, before comparing age distributions of lead SNPs associated with brain phenotypes to null distributions constructed by 10,000 random samples of equal-sized SNP-sets.

For manuscripts utilizing custom algorithms or software that are central to the research but not yet described in published literature, software must be made available to editors and reviewers. We strongly encourage code deposition in a community repository (e.g. GitHub). See the Nature Portfolio guidelines for submitting code & software for further information.

Data

Policy information about availability of data

All manuscripts must include a data availability statement. This statement should provide the following information, where applicable:

- Accession codes, unique identifiers, or web links for publicly available datasets
- A description of any restrictions on data availability
- For clinical datasets or third party data, please ensure that the statement adheres to our policy

Neuroimaging and genetic data from the UK Biobank ([https://www.ukbiobank.ac.uk/) was accessed using accession code 27412.
GWAS summary statistics obtained from the Psychiatric Genomics Consortium [https://www.med.unc.edu/pgc/shared-methods/], and GWAS catalog ().
The Human Genome Dating Dataset (HGD) is available at https://human.genome.dating.

Human research participants

Policy information about studies involving human research participants and Sex and Gender in Research.

Reporting on sex and gender	Self-identified gender was collected upon recruitment by the UK Biobank team. Biological sex was subsequently determined genetically and used for downstream analyses. Cerebellar morphological features were adjusted for main effects of biological sex, as well as for age effects estimated separately for each biological sex, prior to being included in genetic analyses. Genetic analyses were restricted to the 22 autosomal chromosomes.
Population characteristics	For the discovery analysis we included 27,302 non-related White Europeans (age range: 45.1-82.1; mean age: 64.1). 54.9% of the discovery sample was female. For the replication analysis we included 11,264 non-related White Europeans (age range: 46.1-83.7; mean age: 66.8). 46.9% of the replication sample was female.
Recruitment	The participants were obtained from the UK Biobank, which is a population-based cohort, on a voluntary basis. Recruitment procedures are described extensively in the UK Biobank design paper, referenced in the manuscript. the participants are known to be of somewhat above average health.
Ethics oversight	This is analysis of publicly available data. The set-up of the UK Biobank, including description of informed consent and other ethical procedures is extensively described in the UK Biobank design paper, referenced in the manuscript

Note that full information on the approval of the study protocol must also be provided in the manuscript.

Field-specific reporting

Please select the one below that is the best fit for your research. If you are not sure, read the appropriate sections before making your selection.

☒ Life sciences ☐ Behavioural & social sciences ☐ Ecological, evolutionary & environmental sciences

For a reference copy of the document with all sections, see nature.com/documents/nr-reporting-summary-flat.pdf

Life sciences study design

All studies must disclose on these points even when the disclosure is negative.

Sample size	No statistical methods were used to pre-determine sample sizes. We included as much data as we could gather, the sample size is thus based on data availability. After quality control the discovery sample consisted of 27,302 participants and the replication sample consisted of 11,264 participants.
Data exclusions	The study was restricted to participants of European ancestry. We thus excluded 4,818 non-european UK Biobank participants. Participants with the following neurological disorders were excluded from the sample.
Replication	We performed replication analysis in a separate sample of UK Biobank participants (n = 11,264). In order to ensure that the multivariate patterns - and not only single locus associations - replicate, we employed a multivariate procedure, which computes a composite score from the mass-univariate z-statistics (i.e., applying multivariate weights from the discovery sample to the replication sample input data) and then tests for associations between this composite score and genotypes in the replication sample. 12 of the 351 locus lead SNPs could not be tested as they were not available in the replication sample.<br>Attempts at replication were successful: We found that 97% of loci lead SNPs present in both samples replicated at a nominal significance threshold of $p < .05$, and that 74% remained significant after Bonferroni correction for the 339 replication tests conducted. Moreover, 99% of loci lead SNPs showed the same effect direction across discovery and replication samples. Thus, 329 (94%) of the 351 reported loci were replicated.
Randomization	Randomization is not applicable in the current study design. The group assignment for discovery and replication samples were primarily based on data availability (e.g., later released UK Biobank participants were included in the replication sample), and no group assignment that would require randomization was made.
Blinding	The study design did not require blinding (e.g., no case-control design).

Reporting for specific materials, systems and methods

We require information from authors about some types of materials, experimental systems and methods used in many studies. Here, indicate whether each material, system or method listed is relevant to your study. If you are not sure if a list item applies to your research, read the appropriate section before selecting a response.

Materials & experimental systems

n/a	Involved in the study	
☒	☐	Antibodies
☒	☐	Eukaryotic cell lines
☒	☐	Palaeontology and archaeology
☒	☐	Animals and other organisms
☒	☐	Clinical data
☒	☐	Dual use research of concern

Methods

n/a	Involved in the study	
☒	☐	ChIP-seq
☒	☐	Flow cytometry
☐	☒	MRI-based neuroimaging

Magnetic resonance imaging

Experimental design

Design type	Anatomical MRI
Design specifications	Not applicable.
Behavioral performance measures	None

Acquisition

Imaging type(s)	Structural, T1w
Field strength	3T
Sequence & imaging parameters	TR = 2000ms; TE=2.01ms; FA=8 (Three identical scanning sites); employing a Siemens 3T Skyra
Area of acquisition	Whole brain

Diffusion MRI ☐ Used ☒ Not used

Preprocessing

Preprocessing software	MRI data was first processed using the recon-all pipeline in Freesurfer 5.3 (https://surfer.nmr.mgh.harvard.edu). Next, the bias-field corrected T1-images from the FreeSurfer analyses were analyzed using the cerebellum-optimized SUIT-toolbox (https://github.com/jdiedrichsen/suit).
Normalization	Normalization of individual cerebella to the SUIT template was performed using the SUIT-tolbox for SPM ()https://github.com/jdiedrichsen/suit).
Normalization template	The SUIT probabilistic cerebellar template (https://github.com/jdiedrichsen/suit).
Noise and artifact removal	Standard pipelines for anatomical data were applied (Freesurfer recon-all). Euler number was calculated as a proxy of image quality and data from individuals with insufficient image quality were excluded. For the remaining participants, the Euler number was included as a covariate, in order to adjust for effects systematically related to data quality.
Volume censoring	N/A

Statistical modeling & inference

Model type and settings	Multi-variate
Effect(s) tested	Effect of each SNP, across the genome, on a set of 23 cerebellar morphology measures.

Specify type of analysis: ☐ Whole brain ☒ ROI-based ☐ Both

Anatomical location(s)	We used a data-driven decomposition (using non-negative matrix factorization, NNMF) of modulated probabilistic cerebellar grey matter maps (that is, spatial maps where each voxel-value reflects local volume). NNMF yields sparse - and non-negative - parcellations, reflecting cerebellar regions where volume co-varies across participants. We tested parcel-numbers between 2 and 100, and decided on using a 23-parcel solution for the genetic analyses.
Statistic type for inference (See Eklund et al. 2016)	Permutation testing and replication in independent data.
Correction	Bonferonni correction (p=5*10-8)

Models & analysis

n/a	Involved in the study
☒	☐ Functional and/or effective connectivity
☒	☐ Graph analysis
☐	☒ Multivariate modeling or predictive analysis

Multivariate modeling and predictive analysis

Let z_ij be the value of signed test statistic (z-score) calculated from the univariate association test between j-th SNP and i-th phenotype. Let z_j=(z_1j,...,z_Kj) be the vector of z-scores of j-th SNP across K phenotypes. Let Z={z_ij} be the matrix of z-scores, with rows corresponding to SNPs, and columns corresponding to phenotypes. Further, let Z "={z "_ij } be the matrix of z-scores, calculated from association tests on a randomly permuted genotype vector of each SNP. To preserve correlation structure among phenotypes, the permutation was performed only once for each SNP, and the resulting genotype vector was used in association test across all phenotypes.
The MOSTest test statistic, X_j^2, for the j-th SNP is calculated as Mahalanobis norm X_j^2=z_j^T R "^(-1) z_j, where R "is the KxK correlation matrix of Z ". The null hypothesis of the MOSTest is that z_j is distributed as a multivariate normal random variable with zero mean and covariance R ". To compute the theoretical (i.e., under null) p-value of the MOSTest test statistic, we calculated the tail probability that a Chi-square statistics exceeds X_j^2. This probability is given by chi-square distribution with N degrees of freedom, or, equivalently, a gamma distribution, Gamma(K/2,0.5)26. Instead of using theoretical values, we fit the two free parameters of the Gamma(a,b) distribution to the observed distribution of X_j^2 under permutation (shown in Table S4). The p-value of the MOSTest test statistic is then obtained from a cumulative distribution function of the gamma distribution, p_MOST=CDF_gamma(a,b) (z_j^T R "^(-1) z_j).
Controlling for covariates, such as genetic principal components, is done via pre-residualization of all phenotype vectors, i.e. we replace them with the corresponding residual after multiple linear regression of the phenotype vector on the covariates. Additionally, we perform a rank-based inverse normal transformation of the residualized phenotypes, to ensure that z-scores forming the input to MOSTest are normally distributed.
